# Dysregulated repair and inflammatory responses by e‐cigarette‐derived inhaled nicotine and humectant propylene glycol in a sex‐dependent manner in mouse lung

**DOI:** 10.1096/fba.2019-00048

**Published:** 2019-09-13

**Authors:** Qixin Wang, Naushad Ahmad Khan, Thivanka Muthumalage, Gina R. Lawyer, Samantha R. McDonough, Tsai‐Der Chuang, Ming Gong, Isaac K. Sundar, Virender K. Rehan, Irfan Rahman

**Affiliations:** ^1^ Department of Environmental Medicine University of Rochester Medical Center Rochester NY USA; ^2^ Department of Pediatrics and Molecular Toxicology David Geffen School of Medicine Los Angeles Biomedical Research Institute at Harbor‐UCLA Medical Center Torrance CA USA

**Keywords:** dysregulated repair, extracellular matrix remodeling, inflammation, nicotine, propylene glycol

## Abstract

Nicotine inhalation via electronic cigarettes (e‐cigs) is an emerging concern. However, little is known about the acute toxicity in the lungs following inhalation of nicotine‐containing e‐cig aerosols. We hypothesized that acute exposure to aerosolized nicotine causes lung toxicity by eliciting inflammatory and dysregulated repair responses. Adult C57BL/6J mice were exposed 2 hours daily for 3 days to e‐cig aerosols containing propylene glycol (PG) with or without nicotine. Acute exposure to nicotine‐containing e‐cig aerosols induced inflammatory cell influx (neutrophils and CD8a^+^ T lymphocytes), and release of pro‐inflammatory cytokines in bronchoalveolar lavage fluid in a sex‐dependent manner. Inhalation of e‐cig aerosol containing PG alone significantly augmented the lung levels of various homeostasis/repair mediators (PPARγ, ADRP, ACTA2, CTNNB1, LEF1, β‐catenin, E‐cadherin, and MMP2) in a sex‐dependent manner when compared to air controls. These findings were accompanied by an increase in protein abundance and altered gene expression of lipogenic markers (PPARγ, ADRP) and myogenic markers (fibronectin, α‐smooth muscle actin and β‐catenin), suggesting a dysregulated repair response in mouse lungs. Furthermore, exposure to nicotine‐containing e‐cig aerosols or PG alone differentially affected the release of pro‐inflammatory cytokines in healthy and COPD human 3D EpiAirway tissues. Overall, acute exposure to nicotine‐containing e‐cig aerosols was sufficient to elicit a pro‐inflammatory response and altered mRNA and protein levels of myogenic, lipogenic, and extracellular matrix markers in mouse lung in a sex‐dependent manner. Thus, acute exposure to inhaled nicotine via e‐cig leads to dysregulated repair and inflammatory responses, which may lead to airway remodeling in the lungs.

## 1 INTRODUCTION

Electronic nicotine delivery systems (ENDS) have recently emerged as an alternative to cigarette smoking.^1^, ^2^ Electronic cigarettes (e‐cigs) are devices which use heat to aerosolize a small amount of e‐liquid containing nicotine (up to 24‐100 mg/mL) solubilized in propylene glycol (PG) or vegetable glycerin (VG) as a base constituent, humectant.^3^, ^4^ Although e‐cig use, or “vaping,” is perceived as safer than smoking conventional tobacco cigarettes,^5^, ^6^, ^7^ very little is known about the systemic and pulmonary consequences of acute and chronic e‐cig exposure in vivo. With the recent emergence and increasing popularity of multiple devices for the recreational inhalation of non‐combustible nicotine, eg, e‐cigs among youth/adults, understanding the toxic effects of inhaled nicotine is necessary. E‐cig derived nicotine aerosols may alter physiological properties of the cell by binding to its receptors, ie, nicotinic acetylcholine receptors (nAChRs), which are part of the acetylcholine neurotransmitter signaling pathway of the central nervous system,^8^, ^9^ and are also abundantly expressed in fibroblasts and epithelial cells of the lung.^10^, ^11^

We have previously shown that acute exposure to vapors produced by e‐cigs mixed with flavoring chemicals results in lung cellular oxidative stress and inflammatory and toxicity responses.^12^ Inhaled nicotine via e‐cig exposure leads to impaired immune defense against viral and bacterial infections and increases allergen‐induced airway hyper‐responsiveness.^2^, ^13^, ^14^ Studies have shown that nicotine may increase cardiovascular stress and cardiopulmonary responses.^15^, ^16^ Previous studies have also demonstrated that acute exposure of aerosolized nicotine enhances impedance, peripheral airway flow resistance, and oxidative stress among healthy smokers,^17^, ^18^ and airway hyperactivity in lipopolysaccharide‐challenged mice.^19^ Data from other studies indicate that acute exposure to ENDS is capable of delivering so much nicotine to the bloodstream that it can manifest physiological and cardiovascular effects similar to those of tobacco smoking.^20^ However, very limited information is available specifically related to e‐cig derived nicotine (inhaled nicotine)‐induced pulmonary toxicity (eg, inflammation, oxidative stress, and dysregulated repair) in animal models or the toxicological effects of inhaled nicotine aerosols on human airways. Propylene glycol (C_3_H_8_O_2_) is a clear, colorless, oily, synthetic organic solvent used in various applications from the food industry to pharmaceutical preparations. The oral administration of PG is considered relatively safe and therefore it is used as a humectant base in e‐liquids for vaping. However, very little information is available on inhalation toxicity induced by PG alone or in mixture, eg, PG with nicotine, used as e‐liquids in e‐cigs.

We hypothesized that e‐cig aerosols containing PG with or without nicotine induce sex‐dependent lung inflammatory responses and dysregulated repair. We exposed adult C57BL/6J mice to e‐cig aerosols containing PG with and without nicotine using an e‐cig device in order to determine the mechanism underlying the lung pathophysiological consequences (inflammatory and dysregulated repair markers) in our acute model of inhaled nicotine exposure. The inflammatory response to aerosolized nicotine was also examined using an in vitro human 3D EpiAirway™ model/system.

## 2 MATERIALS AND METHODS

### 2.1 Ethics statement

All experiments using animal models were performed in accordance with the standards established by the United States Animal Welfare Act, as set forth by the National Institutes of Health (NIH) guidelines. The Animal Research Committee of the University of Rochester (UCAR) approved the research protocol for mouse experiments included in this study.

### 2.2 Animals

Adult C57BL/6J mice of both sexes were purchased from the Jackson Laboratory, (body weight 25‐32 gms; 14‐16 weeks old) and were housed in the Inhalation Core facility at the University of Rochester for a 1‐week acclimatization period before beginning acute e‐cig exposure.

### 2.3 E‐cig device and e‐liquids PG with and without nicotine

The ENDS and e‐liquids used to perform experiments in this study have been described previously.^21^ In brief, the Joytech eVIC VTC mini ENDS was used for the acute 3 day e‐cig exposure to aerosols containing PG with or without nicotine. The atomizer/coil (0.15 Ω) was purchased from Kanger Tech. All of the remaining components of e‐cigs were purchased from SCIREQ. The atomizer/coil was replaced on a daily basis to prevent overheating of the coil. The e‐liquids, PG and PG with nicotine (25 mg/mL) were purchased from http://xtremevaping.com online store.

### 2.4 E‐cig nicotine exposure (In vivo)

The whole‐body mouse exposure was performed as described previously.^21^ In brief, in vivo e‐cig exposure was performed inside a fume hood using the InExpose e‐cig extension smoking system (SCIREQ). The 3rd generation e‐cig device from Joytech (eVIC VTCmini) was triggered with the help of a stroke controller, which was itself controlled by the Scireq flexiware software (Version 8.0). The e‐cig exposure puff profile and stroke duration were based on realistic topography data from e‐cig users. Exposure temperature, humidity, and percentages of oxygen and carbon dioxide were recorded by the software throughout the exposure using the atmospheric monitoring control module. The puff profile was set at 2 puffs/min (3.3 sec/puff with a 70 mL puff volume for a total of 2 hours exposure time for both PG and PG with nicotine for three consecutive days. The flexiware software controlled generation of e‐cig aerosols initially passes through the condensing chamber, and then via the pump (flow rate of 1.0 L/min) into the mixing chamber where it is diluted with room air before it reaches the exposure chamber. Mice are placed inside the exposure chamber separated by dividers to keep them apart. Simultaneously, the air and e‐cig aerosol mixture from inside the exposure chamber is exhausted out of the chamber by another pump, set at 2.0 L/min. Each day before initializing the e‐cig exposure setup, the flow rates of pumps 1 and 2 (for e‐cig exposure and exhaust, respectively) were calibrated and adjusted using a flowmeter provided by SCIREQ. Mice were divided into air (control), PG, and PG with nicotine groups with equal numbers of males and females. Exposure to e‐cig aerosols was for 3 days, 2 h/d. Air group mice remained in the same room to ensure similar environmental conditions, but were not exposed to PG or PG with nicotine aerosols. The level of plasma cotinine was measured using a commercially available ELISA kit (Calbiotech) which showed cotinine levels of 23.04 ± 4.89 ng/mL in the PG with nicotine exposed group, but showed no detectable cotinine in PG alone or air‐exposed control groups.

### 2.5 Assessment of PM 1.0, 2.5, 4.0, and 10.0 in e‐cig aerosols

Respirable particle concentration and distribution were measured using the DustTrack II 8530 aerosol monitor (TSI, MN). A dilution chamber with an aerosol sampling port was connected to the SCIREQ inExpose exposure chamber (volume equal to approximately 363.3 cubic inch) without mice. A new 0.15 Ω atomizer was used and heating conditions were set at 230°C and 70 W (power settings). Particle sizes, PM 1.0, PM 2.5, PM 4.0, and PM 10.0, were measured in the dilution chamber over a 10‐minute sampling period. Concentrations were measured every 5 seconds by the aerosol monitor and the average concentration was multiplied by the dilution ratio. For e‐cig aerosols containing PG and PG with nicotine, separate dilution chambers were used (8″ × 5.2″ × 6.5″ with a volume of 270.4 cubic inch, and 15″ × 8″ × 8″ with a volume of 960 cubic inch, respectively). Thus, the final averages obtained by the aerosol monitor were multiplied by dilution factors 0.74 and 2.64 for PG and PG with nicotine, respectively.

### 2.6 Assessment of volatile organic compounds (VOC) in e‐cig aerosols

A Q‐Trak 7575 air quality monitor (TSI, MN) with a VOC probe was used to measure the concentration of volatile organic compounds. The probe was calibrated with zero air (CALGAS Cat# 103L‐1) and spanned with isobutylene (CALGAS cat# 103L‐248N‐100). E‐cig e‐liquids containing PG with and without nicotine were aerosolized into the SCIREQ inExpose exposure chamber without mice and the VOC was measured directly at the aerosol outlet. Stable readings of the VOC concentration were recorded three times during the 10‐minute sampling period and the average was calculated.

### 2.7 Human 3D EpiAirway^TM^ tissues (In vitro exposure)

Human 3D EpiAirway^TM^ tissues (AIR‐100) were obtained from MatTek Corporation. The 3D model is a reconstituted and ready‐to‐use tissue model consisting of normal (AIR‐100‐HCF) and diseased (AIR‐100‐D2‐HCF) human‐derived tracheal/bronchial epithelial cells. EpiAirway tissues from healthy and chronic obstructive pulmonary disease (COPD) donors were exposed to e‐cig aerosols containing PG with nicotine or PG alone. Upon arrival, the tissues were returned to culture on 6‐well plates provided by the supplier and then cultured using an air‐liquid interface (ALI) using the provided culture medium (AIR‐100‐ASY) and overnight incubation at 37°C and 5% CO_2_. At the end of the incubation period, the culture medium was aspirated and replaced with fresh pre‐warmed media and EpiAirway tissues were exposed to e‐cig aerosols (containing 25 mg nicotine with PG or PG alone) for 15 minutes using the modified ALI system and the InExpose e‐cig aerosol system (SCIREQ Inc, Montreal, Canada; with a puff topography of 2 puffs/min (3 s/puff, 51 mL/puff) based on e‐cig users puffing topography as reported previously.^22^ Following the exposure, EpiAirway tissues were incubated at 37°C and 5% CO_2_ for another 24 hours. The human 3D EpiAirway tissue inserts were contained in 35‐mm culture dishes along with 900 μL culture medium during the e‐cig aerosol exposure. The conditioned medium was collected and stored at −80°C for the purpose of measuring pro‐inflammatory mediators.

### 2.8 Bronchoalveolar lavage fluid (BALF)

Mice were euthanized via intraperitoneal injection of pentobarbital (100 mg/kg body weight; Abbott Laboratories) 2 hours after the last e‐cig aerosol exposure. The trachea of each mouse was cannulated and the lungs were lavaged three times with 0.6 mL of 0.9% NaCl. The recovered fluid aliquots were pooled. The lavage fluid was centrifuged (1000 *g* for 10 minutes, 4°C) and the cell‐free supernatant was stored at −80°C until further analysis. The BALF cell pellets were each re‐suspended in 1.0 mL of 0.9% sodium chloride, and stained with trypan blue (Cat#15250061, ThermoFisher Scientific) to determine the total cell counts/mL using a TC20TM automated cell counter (Cat#1450102, Bio‐Rad).

### 2.9 Determination of differential cell counts using flow cytometry of BALF

Flow cytometric analysis of immune inflammatory cells was performed using cell type‐specific monoclonal antibodies on 3‐day e‐cig aerosol‐exposed samples. Briefly, 2.0‐4.0 × 10^5^ BAL cells were stained with cell type‐specific markers in 1× PBS for 30 minutes, followed by washing and re‐suspension in 0.1 mL of 1x PBS for analysis. Cell‐specific markers such as LY6B.2 Alexa fluor488‐conjugated antibody for neutrophils (Novus Biologicals Cat# NBP213077AF488), CD8a PE‐cy5‐conjugated antibody for T lymphocytes (BD Biosciences 553034), and F4/80 PE‐conjugated antibody for macrophages (BioLegend Cat #123109) were used. Total macrophage, neutrophil and CD4a^+^ and CD8a^+^ lymphocyte counts in BALF were determined by taking the percentage of each cell type observed and multiplying that by the total cell counts. Flow cytometry data acquisition was performed using a Guava® easyCyte™ flow cytometer (Millipore Sigma) and analyzed using Guava® InCyte™ software.

### 2.10 Measurement of pro‐inflammatory cytokines in the BALF

The levels of pro‐inflammatory mediators in BALF from mice exposed to inhaled e‐cig aerosols containing PG and PG with nicotine and mice exposed to air (control) were analyzed by quantitative cytokine assays using the Bio‐Plex Pro mouse cytokine 23‐plex immunoassay kit (Bio‐Rad Laboratory) according to the manufacturer's instructions.

### 2.11 Measurement of myeloperoxidase (MPO) levels in BALF

Myeloperoxidase activity (expressed as mU/mL) in BALF was measured using OxiSelect™ Myeloperoxidase Chlorination Activity Assay Kit according to the manufacturer's instructions (Cat#: STA‐803, Cell Biolabs, Inc).

### 2.12 Quantification of myogenic and lipogenic markers using qPCR

After exposure to e‐cig aerosols containing PG and nicotine or PG alone for three consecutive days, the lung tissues of the mice were snap frozen and stored at −80°C. One hundred milligrams of frozen lung tissue from each mouse was homogenized in Trizol and total RNA was isolated using the RNeasy kit (Qiagen). The quantity and quality of the isolated RNA was determined spectrophotometrically (ND‐1000, NanoDrop Technologies). One microgram of each RNA sample was reverse transcribed using random primers for detection of key myogenic [fibronectin (*FN1*), α‐smooth muscle actin (*ACTA2*), β‐catenin (*CTNNB1*), and lymphoid enhancing factor‐1 (*LEF‐1*)] and lipogenic [peroxisome proliferator‐activated receptor γ (*PPARg*) and adipocyte differentiation‐related protein (*ADRP*)] markers according to the manufacturer's guidelines (Thermo Fisher Scientific).^23^ Quantitative RT‐PCR was carried out using SYBR gene expression master mixes (Applied Biosystems). Reactions were incubated for 10 minutes at 95°C followed by 40 cycles of 15 seconds at 95°C and 1 minutes at 60°C, and level of mRNA expression was determined using the Invitrogen StepOne System with *TUBA1A* used for normalization. All reactions were run in triplicate and relative expression was analyzed with the comparative cycle threshold method (2^−ΔΔCT^), as recommended by the supplier (Applied Biosystems). Values were expressed as fold changes compared to the corresponding air‐exposed control group. The qPCR primer sequences used are listed below: TUBA1A: forward: 5′‐CTCTCTGTGGATTACGGAAAGAAG‐3′, reverse: 5′‐GGTGGTGAGGATGGAATTGTAG‐3′; FN1: forward 5′‐TCCTGTCTACCTCACAGACTAC‐3′, reverse5′‐GTCTACTCCACCGAACAACAA‐3′; PPARγ: forward: 5′‐CTGGCCTCCCTGATGAATAAAG‐3′, reverse: 5′‐AGGCTCCATAAAGTCACCAAAG‐3′; LEF1: forward: 5′‐GGAAGAGCAGGCCAAATACT‐3′, reverse:5′‐GACTCCTGTAGCTTCTCTCTCT‐3′; ADRP: forward: 5′‐AAGAGCCAGGAGACCATTTC‐3′, reverse: 5′‐CCACGAGACATAGAGCTTATCC‐3′; ACTA2: forward: 5′‐GACTCTCTTCCAGCCATCTTTC‐3′, reverse: 5′‐GACAGGACGTTGTTAGCATAGA‐3′; CTNNB1, forward: 5′‐GAGGACAAGCCACAGGATTAC‐3′, reverse, 5′‐CACCAATGTCCAGTCCAAGAT‐3′.

### 2.13 Pro‐inflammatory cytokine analysis

Following 24 hours of e‐cig aerosol exposure using the ALI approach, the conditioned medium was collected and stored at −80°C for measurement of pro‐inflammatory mediators. The cytokine concentrations in the conditioned media collected 24 hours post‐exposure were measured using human IL‐6, IL‐8 (DuoSet, R&D Systems, Inc), and PGE_2_ (Cayman Chemical) enzyme‐linked immunosorbent assay (ELISA) kits, according to the manufacturer's instructions.

### 2.14 Protein extraction from lung tissue

After sacrifice, lung tissues were blotted dry with filter paper and stored at −80^°^C until further use. Thirty milligrams of lung tissue from each mouse was mechanically homogenized with 0.35 mL of radio immunoprecipitation assay buffer (RIPA) containing a protease inhibitor cocktail (Cat#: 78440, ThermoFisher Scientific), and lung tissue homogenates were kept on ice for 45 minutes to allow for complete cell lysis. This was followed by centrifugation of lysates at 15 000 *g* for 30 minutes. After centrifugation, the supernatant was collected as lung homogenate, aliquoted and stored at −80^°^C for western blot analysis. The total protein concentration of the supernatant was measured with a Pierce BCA Protein Assay Kit (Cat#: 23227, ThermoFisher Scientific) using bovine serum albumin as the protein standard.

### 2.15 Immunoblot analysis

Samples of lung tissue homogenate containing about 20‐30 µg of protein were separated on either a 7.5% SDS‐polyacrylamide electrophoresis gel or a 4%‐15% gradient (SDS) polyacrylamide gel (BioRad) and then transferred onto a nitrocellulose membrane (Amersham). Non‐specific binding sites were blocked with 5% non‐fat dry milk in tris‐buffered saline containing 0.1% Tween 20 for 1 hour at room temperature. Membranes were then probed with specific primary antibodies: PPARγ (1:1,000, #2443, Cell Signaling Technology); LEF‐1 (1:1000, #2286, Cell Signaling Technology); E‐cadherin (1:1000, #3195s, Cell Signaling Technology); β‐catenin (1:1000, ab32572, Abcam); nAChRα7 (1:500, ab216485, Abcam); nAChRα3 (1:500, ab183097, Abcam); Fibronectin (1:1000, ab23750, Abcam); α‐Smooth Muscle Actin (1:1000, ab124964, Abcam); Collagen I α 1 (1:1000, NBP1‐30054, Novus Biologicals); MMP9 (1:1000, ab38898, Abcam); MMP2 (1:1000, ab92536, Abcam); and incubated overnight at 4°C. The membranes were then washed three times for 10 minutes each, and probed with a secondary anti‐rabbit antibody (1:10 000 dilution in 5% non‐fat milk) linked to horseradish peroxidase for 1 hour After incubation, membranes were washed three times for 10 minutes each, and luminescence signals were detected using an enhanced chemiluminescence method (Perkin Elmer, Waltham, MA) following the manufacturer's protocol; images were taken with Bio‐Rad ChemiDoc MP Imaging system (Bio‐Rad Laboratories). The blots were subsequently stripped and equal loading of the samples was determined by re‐probing membranes with β‐actin (1:2500, ab20272, Abcam). Band intensity was determined by densitometry analysis, and normalized using β‐actin as a housekeeping control. Results were expressed as fold change relative to the air‐exposed control group.

### 2.16 Statistical analysis

The statistical significance of the results was calculated using one‐way or two‐way ANOVA, as appropriate, followed by Tukey's multiple comparisons test using GraphPad Prism Software version 7.0. Results are represented as the mean ± SEM unless otherwise indicated. *P* < 0.05 was considered statistically significant.

## 3 RESULTS

### 3.1 E‐cig aerosol particle size and VOC assessment

Using DustTrak II 8530 aerosol monitor, e‐cig aerosols containing PG with nicotine showed higher particle concentrations for all particle size ranges tested when compared to e‐cig aerosols containing PG alone. Additionally, both PG and PG with nicotine aerosols exhibited higher concentrations at PM2.5 (PG: 110.1 mg/m^3^, PG+Nicotine: 887.5 mg/m^3^) and PM 4.0 (PG: 179.7 mg/m^3^, PG+Nicotine: 865.1 mg/m^3^) than PM 1.0 (PG: 81.9 mg/m^3^, PG+Nicotine: 625.4 mg/m^3^) and PM 10.0 (PG: 124.2 mg/m^3^, PG + Nicotine: 208.2 mg/m^3^). Probably, due to the hygroscopic property of PG and agglomeration/humidity or condensation between PG and nicotine, PM, as described above, and VOC showed higher concentration in PG with nicotine than PG alone (PG: 836 ppm, PG+Nicotine: 1023 ppm).

### 3.2 Acute exposure of inhaled nicotine‐containing e‐cig aerosols induces inflammation in mouse lung in a sex‐dependent manner

**Figure 1:**
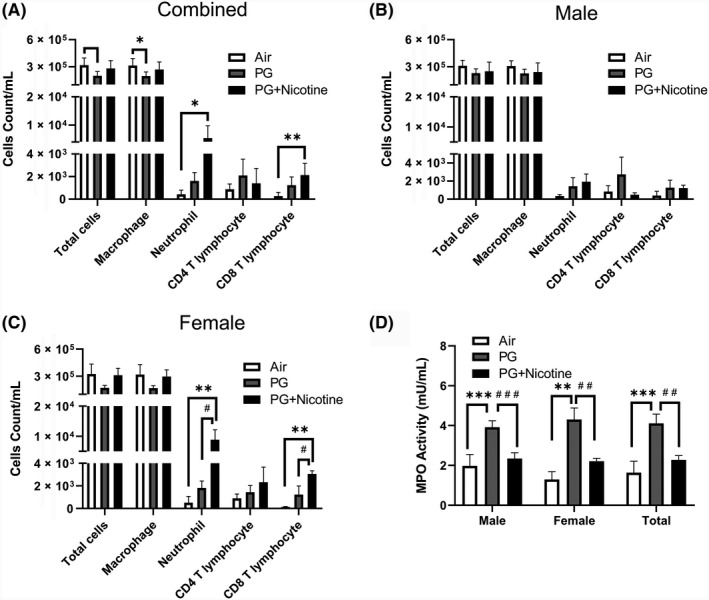
Acute exposure to inhaled e‐cig aerosols containing nicotine increases inflammatory cellular influx in BALF. Mice were exposed to air, PG, and PG with nicotine (PG+Nicotine) for 3 days (2 h/d). Mice were sacrificed 2 hours post final exposure on day 3. Total cell counts per milliliter in BALF were determined by trypan blue staining using Bio‐Rad Tc10 cell counter. Differential cells: F4/80+ macrophages, LY6B.2+ neutrophils, CD4a^+^ T lymphocytes, and CD8a^+^ T lymphocytes were determined using flow cytometry, combined results (A), male (B) and female (C) are presented individually. (D). MPO activity in BALF. Data are shown as mean ± SEM (n = 6/group; equal number of male and female mice were used; n = 3 for male and female groups respectively; *P <0.05, **P < 0.01, ***P < 0.001 significant compared with air‐exposed control; ^#^ *P *< 0.05, ^##^ *P* < 0.01, ^###^ *P* < 0.001 significant compared with PG exposed group.)

We observed that total BALF cell counts in mice exposed to inhaled e‐cig aerosols containing PG alone decreased significantly compared with air‐exposed control (Figure 1). In contrast, there was no change in the total cell count in the PG with nicotine exposed group compared with the air‐exposed control group (Figure 1A). Macrophage counts showed a similar trend (Figure 1A). However, neutrophil count significantly increased in the PG with nicotine exposed group compared with the air‐exposed control group, whereas there was no significant difference in neutrophil counts between the air and PG exposed groups (Figure 1A). Similarly, CD8a^+^ T‐lymphocyte counts showed a significant increase in the PG with nicotine exposed mice, while the PG exposed mice did not show any difference compared to air‐exposed controls (Figure 1A). There was no significant change in CD4a^+^ T‐lymphocyte counts among the experimental groups (Figure 1A). Sex‐dependent analysis of differential cell counts showed significant increases in neutrophil and CD8a^+^ T‐lymphocyte counts in PG with and without nicotine exposed groups compared to air exposed in controls in females, while no significant differences were observed in males (Figure 1B,C). Taken together, the analysis conducted on BALF collected 2 hours after the last exposure (3rd day) to e‐cig aerosols suggests pulmonary inflammation via significant influx of neutrophils and CD8a^+^ T lymphocytes in PG with and without nicotine exposed female mice, but not in males (Figure 1B,C).

Since MPO activity implies the level of neutrophilic inflammation, we next assessed its activity in BALF of mice exposed to e‐cig aerosols containing PG with or without nicotine. Acute exposure to e‐cig aerosols containing PG significantly increased MPO levels compared with both air and PG with nicotine exposed groups (Figure 1D). Surprisingly, PG with nicotine exposed mice showed lower MPO activity than PG only exposed mice even though these mice showed a significant increase in neutrophil counts. Sex‐dependent analysis of BALF MPO activity also showed a similar trend in both males and females, ie, a significantly increased MPO activity in the PG alone exposed group, but a significantly lower activity in the PG with nicotine exposed group (vs. PG exposed group) (Figure 1D).

### 3.3 Exposure to nicotine‐containing e‐cig aerosols induced an influx of inflammatory mediators in mouse lung in a sex‐dependent manner

**Figure 2:**
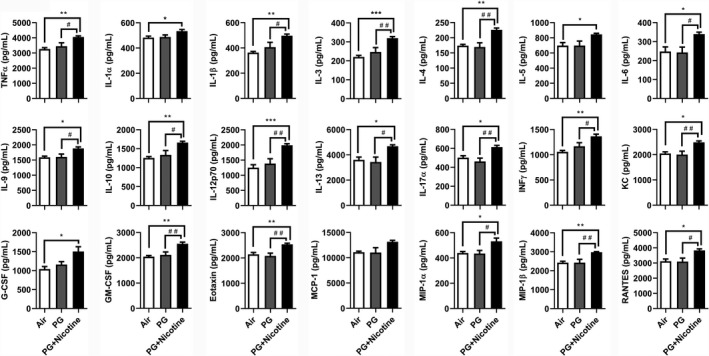
Acute exposure to inhaled e‐cigarette aerosols containing nicotine caused pro‐inflammatory mediator release in BALF. Mice were exposed to air, PG, and PG with nicotine (PG+Nicotine) for 3 days (2 h/d). Mice were sacrificed 2 hours post‐last exposure on day 3. Pro‐inflammatory mediators in the BALF were measured using Bio‐Plex Pro‐mouse cytokine 23‐plex assay kit (Bio‐Rad). Data are shown as mean ± SEM (n = 6/group; equal number of male and female mice were used; *P < 0.05, **P < 0.01, ***P < 0.001, significant compared with air control; ^#^ *P* < 0.05, ^##^ *P* < 0.01, significant compared with PG exposed group.)

To further assess the inflammatory response to inhaled nicotine‐containing e‐cig aerosols in mouse lungs, a panel of cytokines/chemokines was measured in BALF of mice from different experimental groups. The levels of pro‐inflammatory cytokines TNFα, IL‐1α, IL‐1β, IL‐3, IL‐4, IL‐5, IL‐6, IL‐9, IL‐10, IL‐12p70, IL‐13, IL‐17α, IFNγ, KC, G‐CSF, GM‐CSF, eotaxin, MIP‐1α, MIP‐1β, and RANTES were significantly increased in mice exposed to e‐cig aerosols containing PG or PG and nicotine compared with air‐exposed control mice (Figure 2). Note, except for IL‐1α, IL‐5, and G‐CSF, all other pro‐inflammatory cytokines mentioned above were significantly increased in PG with nicotine compared to PG alone group (Figure 2). Furthermore, IL‐2, IL‐12, (data not shown) and MCP‐1 showed no significant changes among the three experimental groups. These data indicate that acute exposure to e‐cig aerosols containing PG with nicotine enhanced the release of pro‐inflammatory mediators in mouse lungs, and was sufficient to elicit an inflammatory response.

Among all of the pro‐inflammatory cytokines/chemokines examined, 15 out of 23 showed significant sex‐dependent effects (Table 1). Of all the cytokines analyzed, TNFα, IL‐3, IL‐4, IL‐9, IL‐12p70, IL‐13, IL‐17α, IFNγ, KC, GM‐CSF, Eotaxin, MIP‐1α, MIP‐1β, and RANTES, showed significant differences between PG alone and PG with nicotine exposed groups in males. Among these, IL‐3, IL‐4, IL‐9, IL‐12p70, IFNγ, GM‐CSF, Eotaxin, and MIP‐1β showed a significant increase in PG with nicotine exposed males compared to air‐exposed controls. Only two cytokines, IL‐3 and KC were significantly increased in PG with nicotine exposed females and males compared to PG alone or air‐exposed controls. Furthermore, only IL‐1β was significantly increased in females exposed to PG with nicotine compared to air‐exposed control, whereas, males did not a show significant change in IL‐1β release in BALF.

**Table 1:** Sex‐dependent effect of pro‐inflammatory cytokine/chemokine release in BAL fluid following acute e‐cig exposure containing PG with and without nicotine

Cytokines/ Chemokines (pg/mL)	Female			Male		
	Air	PG	PG + Nicotine	Air	PG	PG + Nicotine
TNFα	3222 ± 163.8	3739 ± 280.6	4027 ± 98.46	3309 ± 90.47	3154 ± 301.8	4093 ± 122.7**
IL‐1β	345.3 ± 9.323	437.8 ± 52.79	491.5 ± 8.392*	380.3 ± 9.53	374.6 ± 58.3	502.5 ± 25.81
IL‐3	223.4 ± 13.94	279.9 ± 21.92	312.0 ± 6.243*	217.9 ± 11.41	213.7 ± 32.63	327.4 ± 15.19* *^,^* **
IL‐4	182.6 ± 1.340	185.1 ± 21.52	233.3 ± 0	165.6 ± 3.397	154.0 ± 16.45	218.6 ± 11.68* *^,^* **
IL‐9	1623 ± 71.43	1679 ± 163.7	1837 ± 29.79	1556 ± 34.87	1531 ± 107.0	1931 ± 97.09* *^,^* **
IL‐12p70	1262 ± 213.5	1577 ± 185.9	1927 ± 24.56	1238 ± 44.47	1191 ± 244.0	2052 ± 79.32* *^,^* **
IL‐13	3737 ± 348.6	3958 ± 387.3	4702 ± 74.58	3475 ± 312.6	2914 ± 563.8	4658 ± 250.5**
IL‐17α	514.8 ± 45.96	510.9 ± 36.61	624.6 ± 24.66	488.2 ± 60.67	412.5 ± 52.07	603.4 ± 29.16**
IFNγ	1064 ± 47.54	1226 ± 132.2	1306 ± 10.21	1050 ± 42.61	1108 ± 67.07	1422 ± 77.05* *^,^* **
KC	2023 ± 113.2	2180 ± 150.6	2537 ± 68.73*	2076 ± 77.19	1833 ± 189.9	2438 ± 98.43**
GM‐CSF	2056 ± 80.65	2249 ± 177.5	2538 ± 18.11	2036 ± 45.09	1978 ± 147.5	2575 ± 147.2* *^,^* **
Eotaxin	2216 ± 134.9	2205 ± 159.1	2538 ± 33.08	2072 ± 65.32	1961 ± 130.4	2546 ± 88.15* *^,^* **
MIP1α	444.8 ± 16.07	468.8 ± 32.37	557.9 ± 49.35	434.1 ± 18.85	401.3 ± 30.19	505.4 ± 18.41**
MIP1β	2538 ± 98.35	2568 ± 274.4	2975 ± 12.70	2316 ± 67.20	2288 ± 242.0	2979 ± 67.47* *^,^* **
RANTES	3219 ± 287.0	3341 ± 342.6	3794 ± 83.83	3015 ± 128.5	2843 ± 303.9	3863 ± 208.6**

Note

n = 3, equal number of female/male mice were used.

Abbreviations: GM‐CSF, granulocyte‐macrophage colony‐stimulating factor; IFNγ, interferon γ; KC, keratinocyte chemoattractant; MIP1α, macrophage inflammatory proteins 1α; MIP1β, macrophage inflammatory proteins 1β; RANTES, regulated on activation, normal T cell expressed and secreted; TNFα, tumor necrosis factor α.

* *P* < 0.05, significant compared with air group control;

** *P* < 0.05, significant compared with PG exposed group.

### 3.4 Inhaled e‐cig aerosols caused dysregulated repair via altered expression of myogenic and lipogenic markers in mouse lung in a sex‐dependent manner

**Figure 3:**
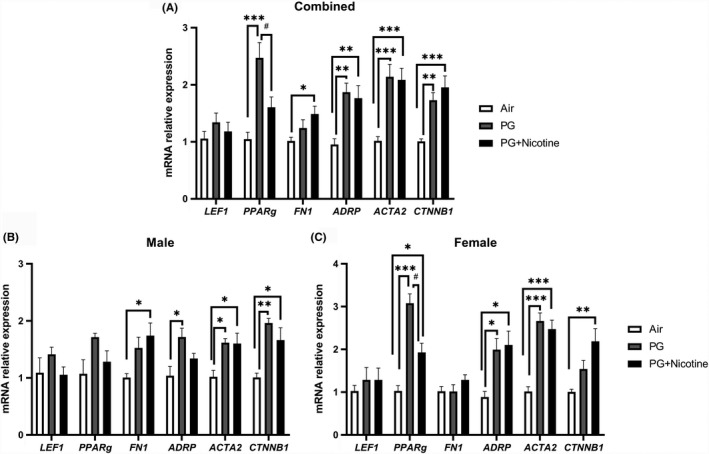
Acute exposure to inhaled e‐cig aerosols containing nicotine alters gene expression of myogenic and lipogenic markers in mouse lungs. Mice were exposed to air, PG, and PG with nicotine (PG+Nicotine) for 3 days (2 h/d). Mice were sacrificed 2 hours post‐last exposure on day 3. RNA was extracted from lung homogenates, and myogenic and lipogenic markers were quantified using qPCR. Gene expression analysis revealed altered mRNA levels of lipogenic and myogenic markers in mouse lungs: Lymphoid enhancer binding factor 1 (*LEF‐1*), Peroxisome proliferator‐activated receptor γ (*PPARg*), Type1 fibronectin (*FN1*), Adipose differentiation‐related protein (*ADRP*), Alpha smooth muscle actin (*ACTA2*), and β‐catenin (*CTNNB1*). Combined results (A), male (B) and female (C) are presented individually. Data are shown as mean ± SEM (n = 8‐9/group; n = 4‐5 male and female mice were used; *P < 0.05, **P < 0.01, ***P < 0.001, significant compared with air control; ^#^ *P * < 0.05, significant compared with PG exposed group)

Using qRT‐PCR, we determined the expression of myogenic and lipogenic markers in lung tissues of mice exposed to e‐cig aerosols containing PG with and without nicotine (Figure 3). Compared to air‐exposed controls, the expression of myogenic markers *ACTA2* and *CTNNB1* increased in the lungs of mice exposed to PG with or without nicotine (Figure 3A); FN1 mRNA levels increased only in mice exposed to PG with nicotine, whereas LEF1 mRNA levels did not change with either intervention (Figure 3A). In contrast, compared to air‐exposed controls, lipogenic differentiation marker PPARγ increased only in mice exposed to PG; surprisingly, in the PG with nicotine exposed group PPARγ mRNA levels were significantly lower compared to PG only exposed group (Figure 3A). Conversely, the gene expression of ADRP, another lipogenic marker, increased significantly on exposure to e‐cig aerosols containing PG with or without nicotine (Figure 3A).

Similar to the effects observed on lung inflammatory mediators, the effects observed on selected lipogenic/myogenic markers in mouse lung were also sex‐dependent (Figure 3B,C). For example, although the lipogenic marker ADRP showed upregulation in PG exposed females and males (Figure 3B,C), in the PG with nicotine exposed group, it was increased only in females. Interestingly, another lipogenic marker, PPARγ was significantly increased in both PG and PG with nicotine exposed females compared to air‐exposed females, while males did not show any significant change (Figure 3B,C). Comparably, myogenic marker, *CTNN1B* was significantly increased in males exposed to PG alone, while the females remained unaffected (Figure 3B,C). Another myogenic marker, *FN1* was only upregulated in males exposed to PG with nicotine, while there was no change in females (Figure 3B,C). Overall, acute e‐cig exposure selectively altered the mRNA levels of lipogenic and myogenic markers in a sex‐dependent manner. Together, these results suggest that inhaled PG with or without nicotine could mediate alterations in the expression of genes involved in myogenic and lipogenic pathways, thereby affecting tissue differentiation and remodeling in mouse lungs.

### 3.5 Inhaled e‐cig aerosol alters abundance of nicotinic acetylcholine receptors, dysregulated repair markers, and extracellular matrix (ECM) proteins in mouse lung in a sex‐dependent manner

**Figure 4:**
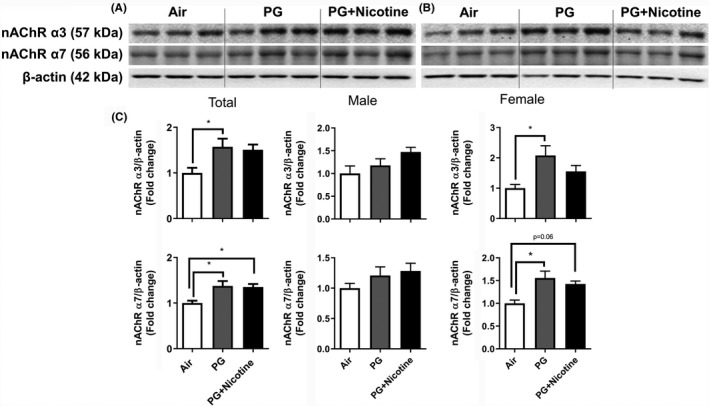
Acute exposure to inhaled e‐cig aerosols containing PG with or without nicotine leads to increased levels of nicotinic acetylcholine receptors in mouse lungs. The protein abundance of nicotinic acetylcholine receptors was measured in lung homogenates using western blotting. β‐actin was used as an endogenous control. Representative blots for nAChR α3 and nAChR α7 levels in air, PG and PG with nicotine exposed male (A) and female (B) mice are shown. (C) The band intensity was measured by densitometry and data are shown as fold change relative to air group control; β‐actin was used as a loading control. Data are shown as mean ± SEM (n = 6/group; equal number of male and female mice were used; n = 3 for male and female groups respectively; *P < 0.05 significant compared with air‐exposed control)

**Figure 5:**
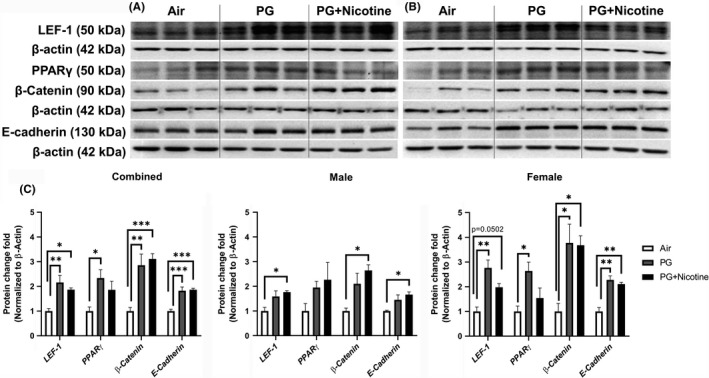
Acute exposure to inhaled e‐cig aerosols containing PG with or without nicotine leads to dysregulated lipogenic/myogenic markers in mouse lungs. The protein abundance of lipogenic and myogenic markers was measured in lung homogenates using western blotting. β‐actin was used as an endogenous control. Representative blots for lymphoid enhancer binding factor 1 (LEF‐1), peroxisome proliferator‐activated receptor *γ* (PPAR*γ*), β‐Catenin, and E‐cadherin levels in air, PG and PG with nicotine (PG+nicotine) exposed (A) male and (B) female mice are shown. (C) The band intensity was measured by densitometry and data are shown as fold change relative to air group control; β‐actin was used as a loading control. Data are shown as mean ± SEM (n = 6/group; equal number of male and female mice were used; n = 3 for male and female groups respectively; *P  < 0.05, **P < 0.01, ***P < 0.001, significant compared with air‐exposed control)

**Figure 6:**
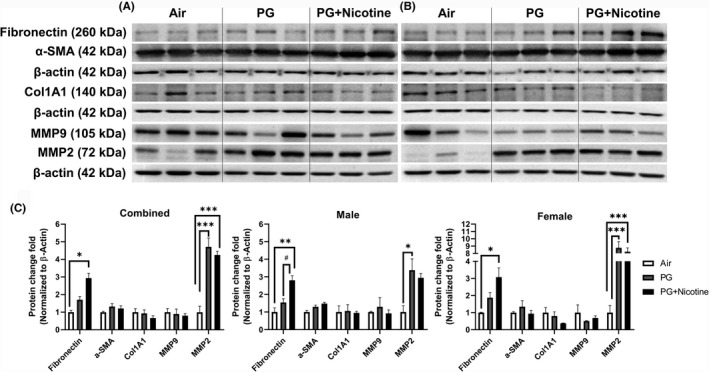
Acute exposure to inhaled e‐cig aerosols containing PG with or without nicotine leads to dysregulated ECM proteins in mouse lungs. The protein abundances of ECM‐related markers were measured in lung homogenates using western blotting. Representative blots for fibronectin, alpha smooth muscle actin (α‐SMA), Type I collagen (COL1A1), MMP9, and MMP2 levels in air, PG and PG with nicotine exposed (A) male and (B) female mice are shown. (C) The band intensity was measured by densitometry and data are shown as fold change relative to air group control; β‐actin was used as a loading control. Data are shown as mean ± SEM (n = 6/group; equal number of male and female mice were used; n = 3 for male and female groups respectively; *P < 0.05, **P < 0.01, ***P < 0.001, significant compared with air‐exposed control; ^#^ *P < *0.05, significant compared with PG exposed group)

We performed immunoblot analysis to assess alterations in protein abundance caused by inhaled e‐cig aerosols containing PG with and without nicotine in mouse lungs. The results revealed that the levels of nicotinic acetylcholine receptor proteins alpha 3 and 7 (nAChRα3 and nAChRα7) increased in both PG with and without nicotine exposed groups compared with the air‐exposed control group (Figure 4). These effects were more robust in females, whereas, in males, only a trend towards significant increases was observed without attaining statistical significance. Notably, PG alone exposure group also demonstrated upregulated nAChRα3 and nAChRα7 protein levels compared to air controls (Figure 4C). When we analyzed protein abundance of myogenic markers, both β‐catenin and LEF‐1, showed significant increases in both PG and PG with nicotine exposed mice compared with air‐exposed controls. However, we did not observe any significant difference between PG and PG with nicotine exposed groups (Figure 5). A similar trend was observed in lipogenic marker PPARγ in mice exposed to PG alone compared to air‐exposed control (Figure 5). Both PG alone and PG with nicotine exposed groups showed significant increases in E‐cadherin levels compared to air‐exposed controls (Figure 5). Additionally, a few ECM‐related proteins, such as FN1 and MMP2 also showed altered protein abundance (Figure 6). Mice exposed to PG or PG with nicotine showed increased MMP2 compared with air‐exposed controls, while FN1 increased only in mice exposed to PG with nicotine (Figure 6). However, other ECM‐related proteins, such as α‐SMA, COL1A1, and MMP‐9, showed no significant differences in the three experimental groups (Figure 6).

As mentioned above, we found sex‐dependent effects in protein abundance of lipogenic and myogenic markers in mouse lung (Figure 5C). Lipogenic markers PPARγ and E‐cadherin increased significantly in females exposed to PG alone, while in males these remained unaffected (Figure 5C). Similarly, myogenic marker β‐catenin also increased significantly only in females exposed to PG alone compared with air group control, while both females and males showed increased β‐catenin protein levels in the PG with nicotine exposed group compared to air control group (Figure 5C). Comparably, ECM‐related protein, MMP2 was significantly increased in both females and males exposed to PG, while only females exposed to PG with nicotine showed an increase compared to air control group (Figure 6C). Another myogenic marker, α‐SMA was significantly increased in males exposed to PG with nicotine, while females remain unaffected (Figure 6C). Overall, acute e‐cig exposure selectively affected protein expression of lipogenic/myogenic and ECM‐related proteins in a sex‐dependent manner. These results corroborate the gene expression of myogenic and lipogenic markers involved in dysregulated repair response following e‐cig exposure.

### 3.6 Exposure to e‐cig aerosols containing nicotine increases pro‐inflammatory cytokine release in human in vitro model of 3D EpiAirway tissues

**Figure 7:**
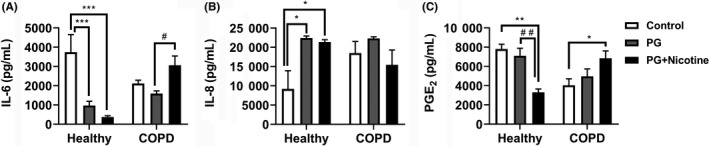
E‐cig aerosols containing nicotine caused pro‐inflammatory cytokine release in healthy and COPD human 3D in vitro model of EpiAirway tissues. Human normal (healthy: Cat.# AIR‐100‐HCF) and diseased (COPD: Cat.# AIR‐100‐D2‐HCF; Donor TBE‐17) 3D models of EpiAirway tissues (MatTek Corp. Ashland, MA) were exposed to e‐cig aerosols containing PG with or without nicotine for 15 minutes (2 puffs/min; 4‐5 s/puff every 25 s) using an air‐liquid interface system, and then transferred to an incubator set at 37°C with 5% CO_2_ for 24 h. Levels of pro‐inflammatory cytokines (A) IL‐6, (B) IL‐8 and (C) PGE_2_ were determined by ELISA in conditioned medium collected 24 hours post‐exposure. Data are shown as means ± SEM (n = 4/group [healthy] and n = 6*/*group [COPD]). *P < 0.05, **P < 0.01, ***P < 0.001 significant compared with control; ^#^ *P < *0.05, ^##^ *P* < 0.01, significant compared with PG exposed group

To determine oxidative stress and inflammatory responses elicited by e‐cig aerosols containing nicotine via in vitro exposure in a 3‐D culture model, we first exposed the human 3D EpiAirway^TM^ tissues (human primary tracheal/bronchial lung epithelial cells) from healthy and COPD donors to e‐cig aerosols. Then, we measured pro‐inflammatory cytokines IL‐6, IL‐8, and PGE_2_ in conditioned media using commercially available ELISA kits. Measurement of IL‐6 release in the conditioned medium collected 24 hours post final exposure revealed that IL‐6 levels decreased significantly in the PG with nicotine and PG alone groups compared to the healthy controls (Figure 7A). However, IL‐6 levels significantly increased in the COPD group exposed to inhaled e‐cig aerosols containing PG with nicotine (Figure 7A). Acute exposure to inhaled e‐cig aerosols containing PG with or without nicotine induced a significant increase in IL‐8 levels in the conditioned media collected 24 hours after the last exposure in the healthy donor group, but showed no significant difference in the COPD group compared to respective controls (Figure 7B). E‐cig aerosol containing PG with nicotine suppressed the release of PGE_2_ in healthy donors compared to controls in the conditioned media collected 24 hours post final exposure (Figure 7C). However, PGE_2_ levels significantly increased in the COPD group in response to inhaled e‐cig aerosols containing PG with nicotine (Figure 7C). These data demonstrate that acute exposure to e‐cig aerosols containing PG and PG with nicotine in a human 3D model of EpiAirway tissue promotes differential release of pro‐inflammatory mediators, such as IL‐6, IL‐8, and prostaglandin E2 (PGE_2_) in the normal (healthy) versus diseased state (COPD).

## 4 DISCUSSION

The use of e‐cigs has gained popularity in western countries including the United States in recent years, and e‐cig usage is increasing among smokers, nonsmokers, women, adolescent and teenagers. The use of e‐cigs is commonly regarded as a safer alternative to conventional cigarette smoking, despite the minimal availability of data on their relative safety. Although extensive literature exists on the acute and chronic effects of cigarette smoke on lung inflammatory and oxidative stress responses, there are very limited studies on the potential biological effects of e‐cig aerosols on possible injurious responses in the lungs. To the best of our knowledge, no study has yet evaluated the differential effects of inhaled aerosols containing PG with and without nicotine on lung inflammation and dysregulated repair responses in a sex‐dependent manner.

In this study, exposure to e‐cig aerosols containing PG with nicotine showed increased inflammatory cell counts in BALF (neutrophils and CD8a^+^ T‐lymphocyte influx) compared to PG and air‐exposed groups. In contrast, 8 weeks of exposure to e‐cig aerosols containing PG with and without nicotine has previously been reported to show reduced BALF total cell count, as well as macrophages along with a modest increase in neutrophils.^24^ Interestingly, after 3 days of e‐cig exposure in male mice, Glynos et al^25^ reported increased total cell count and macrophages, and even higher cellular influx than seen with cigarette smoke exposure. The differences in these results could be due to several reasons including, but not limited to differences in the method of e‐cig exposure, the mouse strain and sexes used, the types of e‐liquids, the concentration of nicotine, and the vendor/commercial sources of e‐liquids used in these studies. We observed sex‐dependent effects in inflammatory cellular influx in BALF after acute e‐cig exposure. Females exposed to PG with nicotine showed significant increase in neutrophils and CD8a^+^ T‐lymphocyte counts compared to the PG and the air‐exposed control group, while no significant differences were found in males in these experimental groups. These data for the first time show sex‐dependent lung inflammatory responses following in vivo exposure to e‐cig aerosols containing PG.

Myeloperoxidase, a peroxidase enzyme, is a biomarker for neutrophilic infiltration and oxidative stress.^26^ Acute exposure to PG alone significantly increased MPO levels measured in BALF, while PG and nicotine combination inhibited these levels. Previously, we and others have shown that acute exposure to waterpipe and cigarette smoke resulted in augmentation of MPO levels in BALF of mice.^27^, ^28^ In another model when nicotine along with lipopolysaccharide (LPS) was used, MPO levels were significantly reduced in the BALF and lung tissue indicating a protective role of nicotine in mouse lungs,^29^, ^30^ which corroborates our data on MPO levels in BALF of the PG with or without nicotine exposed group. Here, for the first time, we report that acute exposure to PG‐containing e‐cig aerosol alone can induce oxidative stress in the lung. Supporting this, vaping without nicotine has been previously shown to result in transient lung inflammation, and gas exchange and pulmonary function abnormalities.^31^ This work suggested that acute vaping of PG/VG aerosol at high wattage with or without nicotine can induce airway epithelial injury and sustained decrement in transcutaneous oxygen tension in young tobacco‐smokers. This highlights that some of the lung effects, which we have seen may be directly related to PG/VG aerosols without nicotine.

Evaluation of inflammatory mediators in BALF showed a significant increase in several pro‐inflammatory cytokines/chemokines following an acute exposure to e‐cig aerosol containing PG with nicotine compared to PG and air control groups in a sex‐dependent manner. These cytokines/chemokines can be produced by a variety of inflammatory cell types including the neutrophils and T lymphocytes, observed to be differentially increased in BALF of different experimental groups.^32^, ^33^ A prior report showed that both 3 days and 4 weeks e‐cig exposure did not alter lung levels of IL‐1β, TNFα, and IL‐6.^25^ In another report of a mouse model, lungs of mothers exposed to e‐cig aerosols with and without nicotine (18 mg/mL) showed increased protein levels of pro‐inflammatory cytokines IL‐1β, IL‐6 and TNFα.^34^ These findings are in agreement with our prior studies which demonstrated that a three days e‐cig (16 mg/mL nicotine) exposure caused a significant increase in pro‐inflammatory cytokines (IL‐1α, IL‐6, IL‐13, and MCP‐1) in BALF.^12^ However, as supported by multiple studies,^35^, ^36^, ^37^, ^38^, ^39^, ^40^, ^41^ it is important to point out that the net effect of e‐cig aerosols‐induced BALF and lung tissue cellular and cytokine/chemokine responses on lung (dys) function must be complex, driven by a multitude of interactions among these components and host responses.

In contrast to the work presented here, prior studies have not specifically examined the effect of sex on cytokine release in acute or chronic e‐cig exposure. However, the studies on sex‐dependent effects of exposure to air pollution in humans (altered inflammatory response to viral infection) and the release of inflammatory mediators following LPS and bleomycin induced injury in vivo^42^, ^43^, ^44^ support our findings. Our results indicate that compared to females, males show increased pro‐inflammatory cytokine release following acute e‐cig exposure. Remarkably, previous studies have also suggested that biological effects of sex hormones play a critical role in lung diseases, such as COPD, interstitial pulmonary fibrosis, and asthma.^45^ In line with our data, immune cells and cytokine/chemokines from BALF have been shown to demonstrate sex‐dependent effects, eg, estrogens by binding to T‐ and B‐cells can affect the release of cytokines, such as IL‐4, IL‐10, and TNF‐α release, which are all potential mediators of lung injury and repair.^45^, ^46^, ^47^ However, a recent study by Larcombe *et al* 24 did not find a significant increase in pulmonary inflammation following 8 weeks of e‐cig aerosol exposure in mouse lungs. This may be attributed to the fact that we used a higher, but commonly found concentration of nicotine (25 mg/mL) in e‐liquids, differences in the genetic backgrounds of the mice used (male/female C57BL/6J vs female Balb/c), and/or possible underlying inherent mechanistic differences between the models studied (cigarette‐like e‐cig vs 3rd generation device for e‐cig vaporization).

It is thought that nicotine exposure by any route results in pulmonary toxicity and other respiratory health problems. For example, nicotine promotes airway remodeling via its receptor in airway smooth muscle cells.^48^ Small airway remodeling plays a pivotal role in the development of emphysema^49^, ^50^, ^51^ and myofibroblast differentiation.^52^ We determined whether e‐cig aerosol containing PG or PG with nicotine causes dysregulated tissue differentiation/repair in the lung via an alteration in the expression of lipogenic/myogenic markers. It is well established that mesenchymal‐epithelial cell signaling pathways are central to maintaining homeostasis for normal lung repair/injury responses.^53^ Nicotine disrupts cellular homeostasis by inducing epithelial‐mesenchymal transition (EMT) through Wnt/β‐Catenin signaling pathways in human airway epithelial cells^54^ and by inhibiting TGFβ‐induced myofibroblast differentiation, implicating a defective fibroblast‐myofibroblast axis.^10^ Importantly, nicotine also disrupts specific epithelial‐mesenchymal paracrine signaling pathways, resulting in pulmonary interstitial lipo‐to‐myofibroblast trans‐differentiation that alters pulmonary development and function.^23^, ^55^, ^56^

A recent study showed an additional role of nicotine‐induced dysregulated growth factors and microRNAs that contribute to the development of pulmonary fibrosis, possibly via altered target genes involved in myogenesis.^57^ We found significantly altered sex‐dependent expression of both lipogenic/myogenic and ECM remodeling markers in the lungs of the acute e‐cig exposed mice. Based on findings from this acute e‐cig exposure model, we speculate that chronic exposure of PG and/or PG with nicotine would also cause lung remodeling as a result of altered lipogenesis/myogenesis, including the activation of ECM signaling. Additionally, supporting our previous observations, acute exposure of e‐cig containing PG or PG with nicotine increased protein abundance of nAChRα7 and nAChRα3, suggesting the involvement of these receptors in e‐cig induced lung remodeling.

In the human 3D EpiAirway tissue model, we found that nicotine significantly downregulated IL‐6 secretion by tissues from healthy subjects, while it increased by tissues from COPD subjects. This is in line with nicotine‐mediated inhibition of IL‐6 production in human brain astrocytes in a dose‐dependent manner.^58^ PG with or without nicotine induced significant IL‐8 release by both healthy and COPD donors compared to untreated controls, while no changes in PGE_2_ in any of the groups were observed. These data suggest that e‐cig aerosols containing nicotine can affect the repair potential of primary human EpiAirway cells corroborating a previous study by Sundar et al^59^ who reported that exposure to e‐cig aerosols elicited inflammatory responses in a 3D human EpiGingival tissue model. The increased levels of pro‐inflammatory cytokines (IL‐6 and IL‐8) may cause remodeling of the ECM in the lungs of COPD patients via exposure to e‐cigs containing nicotine. Recent data also show that acute inhalation of e‐cig aerosols alters small airway and macrophage biological responses in normal individuals.^60^ Overall, we show that acute exposure of e‐cig aerosols containing PG with or without nicotine differentially affects inflammatory responses in healthy and COPD 3D models of human EpiAirway tissues.

It is possible that PG inhalation alone, even for a short period, can elicit lung toxicity by triggering inflammatory responses.^61^ This may be due to VOCs of PG as seen in our findings. One may also wish to investigate the potential toxic effects of thermal decomposition products of e‐cig device components that are generated and how these products can affect/modulate the toxicity of the aerosols produced. However, we did not characterize the specific component(s) of e‐cig aerosols that actually drive(s) the effects observed by us; for example, whether it is nicotine, PG, decomposition products of PG, nicotine, or delivery devices, oxidizing radicals, or a combination of these factors. Although differential pulmonary effects (inflammation, cytokine release, mucin production, mucociliary clearance, global DNA methylation, and overall lung function) following acute and chronic e‐cig exposures to mice have been observed,^24^, ^25^, ^34^, ^38^, ^51^, ^62^ further studies are required to understand the exact mechanisms involved. While we await those studies, based on our data we caution for the possibility of inhaled PG/VG or any oil (MCT) droplets‐induced inflammatory pneumonitis that needs to be carefully considered in any e‐cig user presenting with respiratory symptoms.

In conclusion, this study is the first to investigate the sex‐dependent effects of inhaled e‐cig aerosols containing PG with or without nicotine on mouse lungs. Taken together, our data suggest that acute exposure to inhaled e‐cig aerosols is sufficient to elicit sex‐dependent inflammatory responses and dysregulate lipogenesis/myogenesis processes in the mouse lung, as evidenced by the increased numbers of neutrophils and CD8a^+^ T lymphocytes, increased pro‐inflammatory cytokine release, and altered expression of myogenic/lipogenic markers at the mRNA and protein levels. We cannot rule out the additional chronic/long‐term consequences e‐of cig exposure, particularly since e‐cig aerosols also contain toxic metals that can manifest significant cardiopulmonary and systemic effects following chronic e‐cig vaping. Future studies employing chronic exposure are required to gain better insights into pulmonary toxicity with e‐cig use.

## CONFLICT OF INTEREST

The authors have declared that no conflict of interest exists.

## AUTHOR CONTRIBUTIONS

QW, NAK, IKS, IR conceived and designed the experiments; QW, NAK, TM, GRL, SRM, TDC, IKS conducted the experiments; QW, NAK, TM, GRL, SRM, TDC, MG analyzed the data; QW, NAK, TM, SRM, IKS, VKR, IR wrote and revised/edited the manuscript.
